# Nitrogen-Doped Porous Carbon Derived from Biomass Used as Trifunctional Electrocatalyst toward Oxygen Reduction, Oxygen Evolution and Hydrogen Evolution Reactions

**DOI:** 10.3390/nano10010076

**Published:** 2019-12-31

**Authors:** Chinnusamy Sathiskumar, Shanmugam Ramakrishnan, Mohanraj Vinothkannan, Ae Rhan Kim, Srinivasan Karthikeyan, Dong Jin Yoo

**Affiliations:** 1 Centre for Nano and Soft Matter Science, (CeNS), Jalahalli, Bengaluru-560013, India; sathischem85@gmail.com; 2 R&D Education center for whole life cycle R&D of fuel cell systems, Jeonbuk National University, Jeollabuk-do 54896, Korea; rammtech09@gmail.com (S.R.); vinothkannanram@gmail.com (M.V.); 3 R&D Center for CANUTECH, Business Incubation Center, Department of Bioenvironmental Chemistry, Jeonbuk National University, Jeollabuk-do 54896, Korea; 4 Department of Chemistry, Chikkanna Government Arts College, Tirupur-641502, Tamil Nadu, India; environkarthi@gmail.com; 5 Department of Life Science, Graduate School of Department of Energy Storage/Conversion Engineering, and Hydrogen and Fuel Cell Research Center, Jeonbuk National University, Jeollabuk-do 54896, Korea

**Keywords:** golden shower pods biomass, N-doped porous carbon, oxygen reduction reaction, oxygen evolution reaction, hydrogen evolution reaction

## Abstract

Tremendous developments in energy storage and conversion technologies urges researchers to develop inexpensive, greatly efficient, durable and metal-free electrocatalysts for tri-functional electrochemical reactions, namely oxygen reduction reactions (ORRs), oxygen evolution reactions (OERs) and hydrogen evolution reactions (HERs). In these regards, this present study focuses upon the synthesis of porous carbon (PC) or N-doped porous carbon (N-PC) acquired from golden shower pods biomass (GSB) via solvent-free synthesis. Raman spectroscopy, X-ray diffraction (XRD) and X-ray photoelectron spectroscopy (XPS) studies confirmed the doping of nitrogen in N-PC. In addition, morphological analysis via field emission scanning electron microscopy (FESEM) and transmission electron microscopy (TEM) provide evidence of the sheet-like porous structure of N-PC. ORR results from N-PC show the four-electron pathway (average *n* = 3.6) for ORRs with a Tafel slope of 86 mV dec^−1^ and a half-wave potential of 0.76 V. For OERs and HERs, N-PC@Ni shows better overpotential values of 314 and 179 mV at 10 mA cm^−2^, and its corresponding Tafel slopes are 132 and 98 mV dec^−1^, respectively. The chronopotentiometry curve of N-PC@Ni reveals better stability toward OER and HER at 50 mA cm^−2^ for 8 h. These consequences provide new pathways to fabricate efficient electrocatalysts of metal-free heteroatom-doped porous carbon from bio-waste/biomass for energy application in water splitting and metal air batteries.

## 1. Introduction

The current global-energy crisis has provoked researchers to investigate alternative energy storage and conversion systems that should be environmentally benign, inexpensive and highly efficient [1]. The electrochemical oxygen reduction reaction (ORR), oxygen evolution reaction (OER) and hydrogen evolution reaction (HER) are pivotal to next-generation energy production, storage and conversion systems, include water splitting [2,3] fuel cells [4,5,6,7,8] and metal–air batteries [9,10,11]. In this regard, it is essential to develop novel electrocatalysts applicable to ORRs, OERs and HERs with the desired catalytic properties, such as more abundance, higher efficiency, long-term durability and eco-friendliness [12,13]. To date, platinum (Pt) is a state-of-the-art electrocatalyst for ORRs and HERs, while RuO_2_ and IrO_2_ are being served as the most effective electrocatalysts for OERs. Unfortunately, the high electrocatalytic activity of Pt is accompanied with several disadvantages, including high costs, sluggish kinetics, over potential and poor operational durability. Similarly, catalysts for OERs (IrO_2_ and RuO_2_) suffer due to high cost and the lack of long-term stability [14,15]. All these implications greatly hinder the efficiency of energy storage and conversion systems and their large scale applications. Hence, researchers have aimed to replace those aforementioned noble electrocatalysts by low-cost, large surface area and earth-abundant materials for ORRs, HERs and OERs [16]. On the other hand, non-noble materials such as metal oxides (α-Fe_2_O_3_, Co_3_O_4_/C and Mn_3_O_4_), metal phosphates (Co, Fe, Ni and Mn) and dichalcogenides (MoS_2_ and CoS_2_), have been investigated as electrocatalysts in ORRs, OERs and HERs [17,18,19,20,21,22]. Still, these catalysts have the disadvantages of toxic precursor materials, and the production of toxic wastes in the form of hydroxides, oxides and metal complexes. To conquer the limitations given by metal catalysts, various metal-free catalysts, in specific, graphene, carbon nanotubes and porous carbons, are attracting more attention to use in ORRs, OERs and HERs due to large surface area, high efficiency, good stability, better electrical and electrochemical properties and better tolerance in alkaline and acidic media [23]. However, sophisticated synthetic strategy (chemical vapor deposition (CVD), epitaxial graphene growth, the arc discharge method and laser ablation), labor, cost and time consumption, low yield and purity of precursors limits the usage of graphene and carbon nanotubes. By comparison, porous carbons can be easily synthesized from earth abundant carbon-rich bio-wastes, such as coffee beans [24], banana peels [25], waste soybean dregs [26], kidney beans [27], orange peels [28], soybeans [29] and pomelo peels [30], via cost effective and solvent-free methods. Accordingly, porous carbon-based electrocatalysts are attractive from their low-cost, easy accessibility, scalability and environmental friendliness.

Heteroatoms, such as nitrogen (N), sulfur (S), phosphorous (P), fluorine (F) and boron (B), doped porous carbons receive pervasive attention owing to their accelerated electron transfer and electrocatalytic properties, resulting in improvement in ORRs, HERs and OERs [27,31,32]. For instance, Yang et al. [33] synthesized the N-doped hierarchical structure of porous carbon foam (NCF) from cicada sloughs and used as efficient electrocatalysts for ORRs and OERs. NCF shows better overall oxygen electrode activity of 0.770 V (*ΔE* = *E*_OER 10_ - *E*_ORR1/2_) at alkali medium and nitrogen reduction reaction. Huang et al. [34] synthesized nitrogen and boron dipolar doped nanocarbon which exhibited the highest activity and stability for this ORR in alkaline solutions. ‘Chen et al. [35] synthesized N-doped porous carbons (NCS) from the *Typha orientalis* biomass plant and studied its ORR activity in alkali and acid medium, and these NCS show better catalytic activity, stability and better methanol tolerance. However, synthesis of NCS has used multi-step processes such as the hydrothermal method and freeze drying followed by annealing with NH_3_. Again, the synthesis strategy reported in the aforementioned literatures is multi-step, and it should be reduced to a facile one step and a cost-effective method. In this regard, synthesis of N-doped porous carbon (N-PC) from biomass via solvents that have been free pyrolyzed with urea is more suitable method. In this study, we presented the cost-effective synthesis of PCs and N-PCs from relatively abundant, natural, renewable golden shower pod biomass (GSB) via a solvent-free method (pyrolysis with urea). The key factor is the doping of N in a porous carbon skeleton, which alters the electronic and chemical structure of the PC and improves the efficiency of the electrochemical properties. 

The synthesized PC and N-PC catalysts were characterized with various analytical methods include XRD analysis, Raman and morphology analysis. The catalytic activity of N-PCs for ORRs, OERs and HERs was examined in alkaline medium to determine the suitability of the catalyst for energy storage and conversion applications. The N-PC catalysts show robust tri-functional catalytic performance toward HERs, OERs and ORRs in alkaline electrolyte. The ORR results from N-PC show the four-electron pathway (average *n* = 3.6) of ORR with a half-wave potential (E_1/2_) of 0.76 V and Tafel slope of 86 mV dec^−1^. The OER and HER of the N-PC@Ni catalyst shows better over potential values of 314 and 179 mV at 10 mA cm^−2^, and it shows better stability over 8 h at 50 mA cm^−2^.

## 2. Experimental Section

### 2.1. Materials

Analytical grade concentrated hydrochloric acid (HCl, 38%), urea (CH_4_N_2_O, 99%), potassium hydroxide (KOH, 98%) and polyvinylidene fluoride (PVDF) were purchased from Merck Chemicals (Bengaluru, India). Golden shower pod biomass (GSB) was collected from local premises (Bengaluru, India).

### 2.2. Synthesis of Porous Carbon from GSB

GSB was purified before being used as a carbon precursor. First, the pods of GSB were washed by deionized (DI) water and then dried in an oven at 90 °C for 24 h. The dried GSB was pyrolyzed in a tubular furnace at 800 °C for 2 h in an Argon (Ar) atmosphere with a 20 mL min^−1^ flow rate and its heating rate was 5 °C min^−1^. The resultant carbonized sample was stirred with 1 M KOH for 24 h to remove impurities. Subsequently, the mixture was washed with 1 M HCl and DI water until it attained the neutral pH, and then it was dried at 110 °C for 12 h [36].

### 2.3. Synthesis of N-PC 

**Figure 1:**
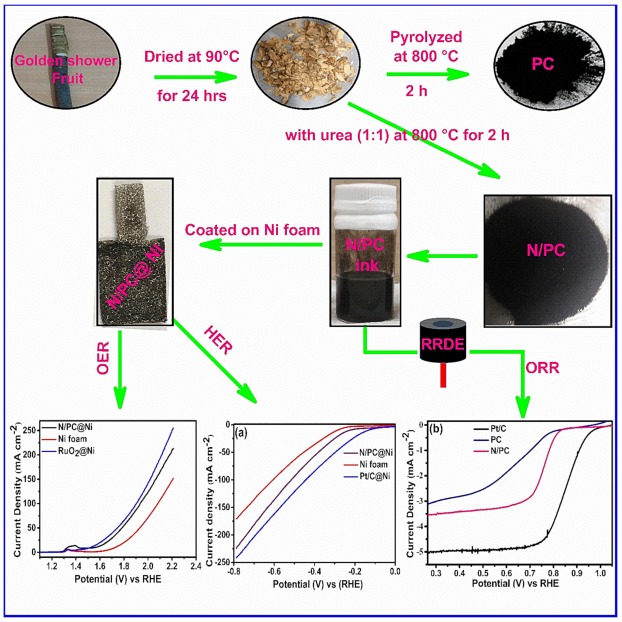
Schematic representation of the synthesis of N-doped porous carbon (N-PC) from golden shower pods biomass (GSB) and its electrochemical activity. (Abbreviations: porous carbon (PC), N-doped porous carbon (N-PC), ring rotating disc electrode (RRDE), oxygen evolution reaction (OER), hydrogen evolution reaction (HER) and oxygen reduction reaction (ORR)).

N-PC was prepared by treating the dried GSB powder with urea via a single step process. Dried GSB powder and urea (1:1 weight ratio) were grained mechanically for 1 h and pyrolyzed at 800 °C under the Ar atmosphere for 2 h. The resultant N-PC was washed by DI water to remove any unreacted CH_4_N_2_O and dried at 80 °C for 12 h. Figure 1 illustrates the detailed synthesis process of N-PC from *GSB* [37].

## 3. Characterizations

The diffractograms were recorded using a PANanalytical X’pert Pro X-ray diffractometer (XRD) (SmartLab, Rigaku, TX, USA). Field emission scanning electron microscopy (FESEM) was performed on an TESCAN-MIRA3 LMH (Brno, Czech Republic). Transmission electron microscopy (TEM) was captured with an FEI Tecnai T 20 U-Twin (Hillsboro, OR, USA). Raman spectra were performed using a Lab RAM HR (Horiba Jobin Yvon) (Horiba SAS, Villeneuve d-Asq, France) with 632.8 nm laser excitation. X-ray photoelectron spectra (XPS) analysis was carried-out using a Kratos Axis Ultra (Kratos Analytical Ltd., Manchester, UK). The surface area and pore diameters were analyzed with a Quantachrome ASIQ (Boynton Beach, FL, USA) win surface area analyzer.

### 3.1. Electrochemical Measurements for ORR

Electrochemical measurements were probed on CHI7001E instrumentation equipped with a three-electrode electrochemical cell. An N-PC-modified glassy rotating disk electrode (RRDE) was employed as a working electrode, and Hg/HgO and a graphitic rod were served as the reference and counter electrodes, respectively. For catalyst slurry preparation, 10 mg of N-PC, was dispersed in isopropyl alcohol (IPA) (0.25 mL) and DI water (0.75 mL) for 20 min. Then, 20 μL of Nafion (5 %) was added as a binder, and the materials were further sonicated for 30 min. 

The as-prepared slurry (5 μL) was coated onto the RRDE electrode (active area around 0.050 mg cm^−2^). All the catalysts were underwent cyclic voltammetry (CV) tests at a scan rate of 10 mV s^−1^ from 0.2 to 1.2 V (vs the reversible hydrogen electrode (RHE)) in an O_2_-saturated 0.1 M KOH electrolyte. Linear sweep voltammetry (LSV) tests were carried out between the potential ranges from 0.2 to 1.2 V (vs RHE). Long-term stability tests for the N-PC and Pt-C chronoamperometry tests were carried out for 10 h, at −0.3 V (vs. Hg/HgO). All recorded potentials were converted to vs. RHE with 0.1 M KOH through the given equation (1).

*E*_(RHE)_ = *E*_(Hg/HgO)_ + 0.89 *V* (1)

### 3.2. Electrochemical Measurements for OER and HER

The N-PC catalyst ink was prepared as follows. Firstly, carbon black, polyvinylidene fluoride (PVDF) and N-PC (1:1:8 weight ratio) were thoroughly grinded together with *N*-methyl-2-pyrrolidone solvent. The catalyst ink (~3.1 mg of catalyst) was deposited onto the Ni foam (1 × 1 cm^2^) and dried at 80 °C for overnight, and was then used as a working electrode. RuO_2_, PC and Pt-C electrodes were also fabricated with a similar procedure, and their OER and HER activities were compared with the N-PC electrode. Three-electrode electrochemical cell setup was utilized to measure the electrocatalytic behavior of prepared electrodes. One out of N-PC@Ni, RuO_2_@Ni, PC@Ni, Pt-C@Ni or the bare Ni foam electrode was used as a working electrode, Ag/AgCl and a graphitic rod were served as reference and counter electrodes, respectively in 1 M KOH aqueous electrolyte solution. LSV measurements were recorded with a scan rate of 1 mV s^−1^ for OERs and HERs. The long-term durability measurements for OERs and HERs were evaluated by chronopotentiometry at a current density of 50 mA cm^−2^ and −50 mA cm^−2^ respectively, for 8 h. All recorded potentials were converted to vs. RHE with 1 M KOH through the given equation (2).

*E*_RHE_ = *E*_Ag/AgCl_ + 1.024 *V* (2)

## 4. Results and Discussion

### 4.1. Morphological Features

**Figure 2:**
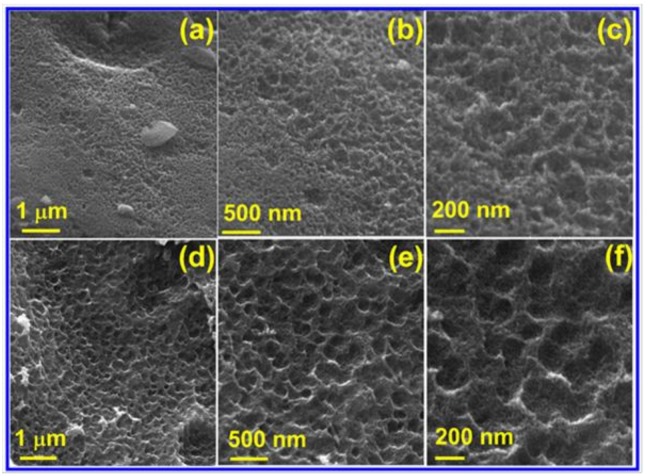
FESEM images of (**a**–**c**) PC and (**d**–**f**) N-PC.

The morphologies of PC and N-PC are presented in Figure 2a–f. Pristine PC shows the morphology of a spongy and loose-like structure, in which a widespread dispersion of meso- and micropores are observed (Figure 2a–c). In contrast, N-PC shows the morphology of well-defined pores with uniform dispersion, where continuous macropores (pore size ranges is 160–210 nm) were observed in the form of 3D networks. This is because of the liberation of gas molecules during the thermal decomposition of urea and GSB throughout the pyrolysis process at 800 °C. Such 3D porous configuration is more beneficial for the adhesion of electrolyte with N-PC, which reduces the three-electrode cell resistance during those ORRs, OERs and HERs. Besides, the N doping facilitates the electron transfer kinetics in the system. To estimate the distribution of N across the PC surface, energy-dispersive analysis X-ray (EDAX) mapping analysis was performed.

**Figure 3:**
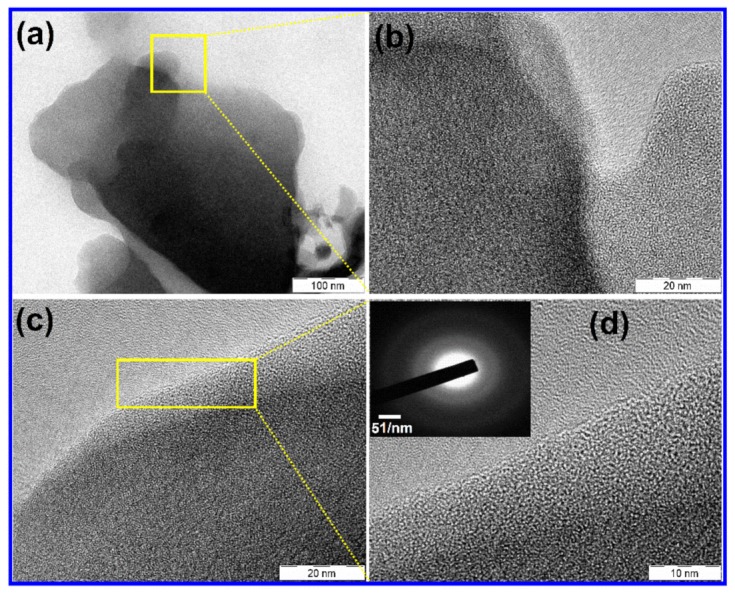
TEM images of (**a**–**d**) N-PC.

It can be seen form Figure S1c that the N was spread spaciously over the surface of the PC; hence rapid electron transport kinetics can be anticipated. To get further understanding on morphology, TEM images of N-PC with various magnifications are provided in Figure 3a–d. N-PC exhibits few layers of graphitic sheet with a wavy shape as a result of N doping (Figure 3a). Figure 3b–d reveals the high-resolution TEM images that demonstrate the distinguished graphitic single layer of N-PC. Selected-area electron diffraction (SAED) of the N-PC is observed to identify the crystallinity of the material (Figure 3d, inset). It is found that the amorphous behavior of N-PC as a result of N doping is as consistent with the literature [38].

### 4.2. Structural Features

**Figure 4:**
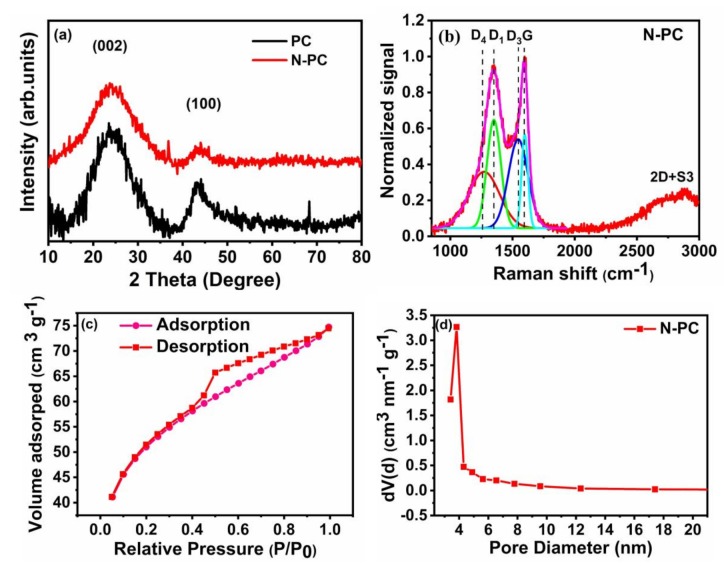
(**a**) XRD pattern and (**b**) Raman spectrum of N-PC with a deconvolution of the D/G spectral region (**c**) nitrogen adsorption/desorption isotherm and (**d**) Barret–Joyner–Halenda model (BJH) pore size distribution plot of N-PC.

The XRD pattern (Figure 4a) of PC and N-PC shows wider carbon (002) and (100) peaks at ~23.6° and ~44.6°, respectively. The characteristic (002) planes of PC and N-PC have a similar interlayer spacing of 0.37 nm. The Raman spectrum of the PC and N-PC were reordered in order to find the structural defect and chemical composition, which can influence their electrocatalytic performance of the ORR and OER/HER. The figure shows the deconvoluted spectrum of the PC (Figure S2) and N-PC (Figure 4b), whereas the samples show two major peaks at ~1595 cm^−1^ and at ~1352 cm^−1^, corresponding two G band D (defect A1) and other deconvoluted two peaks at ~1504cm^−1^ (D3 band) and at 1205 cm^−1^ (D4 band) are corresponding to presence of amorphous carbon and impurities or polyenes, respectively. Further additionally broad peaks were obtained at 2700–3000 cm^−1^, belong to the 2D+S3, which is the mode for out of plane vibration of single or few layer graphene sheet. Whereas, the calculated intensity ratios of I_D1_/I_G_ for N-PC and PC are 1.16 and 0.94 respectively, which confirms the presence of amorous carbon with monocrystalline graphite [39,40]. 

**Figure 5:**
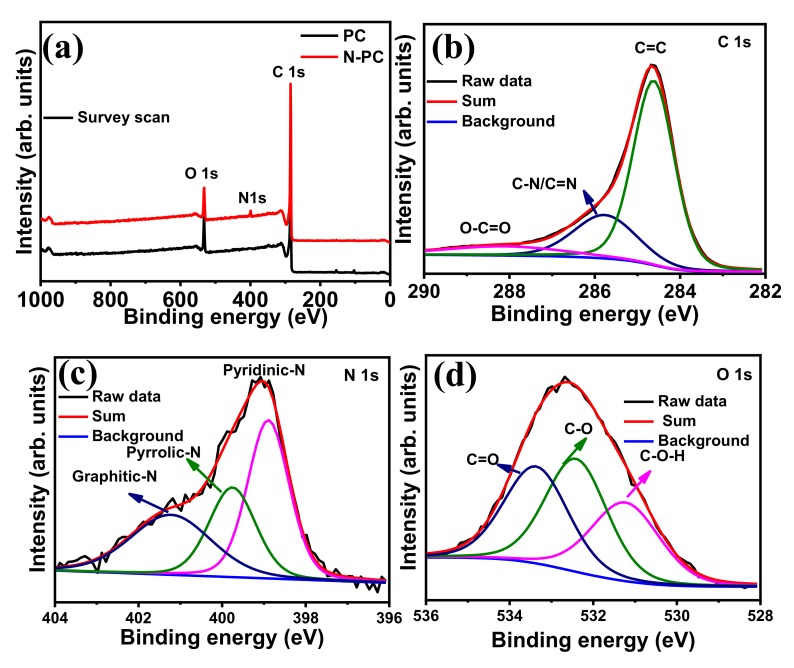
(**a**) XPS survey spectrum of N-PC; deconvoluted XPS spectra of (**b**) C 1s, (**c**) N 1s and (**d**) O 1s corresponding to N-PC.

The N_2_ adsorption/desorption isotherm and type-IV adsorption isotherm for N-PC are provided in Figure 4c,d, respectively. The isotherm curve at low relative pressure (*P/P_0_* < 0.15) is indicative of micropores, and the curve at the intermediate pressure (*P/P_0_* 0.5–0.9) indicates the existence of a highly mesoporous structure in N-PC. The Brunauer-Emmett-Teller (BET) surface area of N-PC as calculated from the isotherm is 839 m^2^ g^−1^. The BJH pore size distribution indicates that the pore diameters are in the range of 2 to 4 nm (Figure 4d), which clearly suggests the existence of meso- and micropores in N-PC. The high surface area of N-PC could enhance the electrocatalytic activity toward the ORR, OER and HER, due to the presence of more active sites. XPS survey spectra of N-PC and PC show C 1s, N 1s and O 1s signals that correspond to C, N and O presence in the sample (Figure 5a). To acquire a deep understanding regarding individual elements in N-PC, the high-resolution spectra of C 1s, N 1s and O 1s were recorded. As shown in Figure 5b, the deconvoluted XPS spectrum of C 1s displays three major peaks: (i) 284.6 eV (C=C and/or C–C) indicates the existence of graphitic carbon, (ii) 285.8 eV is due to the presence of C–N/C=N bonds and (iii) 288.6 eV arises from the O–C=O bonds [41]. Again, the deconvoluted XPS spectrum of N 1s has three peaks at 398.9, 399.7 and 401.2 eV assigned to pyridinic-N, pyrrolic-N and graphitic-N, respectively (as seen in Figure 5c) [38]. The areas of three nitrogen peaks indicate that 30.9%, 42.3% and 26.8% of nitrogen are present in the form of graphitic-N, pyridinic-N and pyrrolic-N, respectively. Similarly, the deconvoluted XPS spectrum of O 1s (Figure 5d) shows three peaks at 531.2, 532.4 and 533.4 eV, corresponding to H–O–C, O–C and O=C respectively. Of particular interest are graphitic-N and pyridinic-N to enhance the activity of the ORRs, OERs and HERs because of their high binding nature with O_2_ [42]. Li et al. [43] found that pyridinic-N in 3D flower-like, N-doped carbon can promote ORR activity via a four-electron transfer pathway. 

Experimental and theoretical studies reported in other literatures indicates that pyridinic-N and graphitic-N boost-up the ORR, OER and HER activities significantly in relation to pyrrolic-N [13,44,45]. Further addition the deconvoluted XPS spectrum of PC (C 1s and O 1s), as shown in Figure S3a,b.

### 4.3. Electrochemical Studies 

#### 4.3.1. ORR Activity and Durability

**Figure 6:**
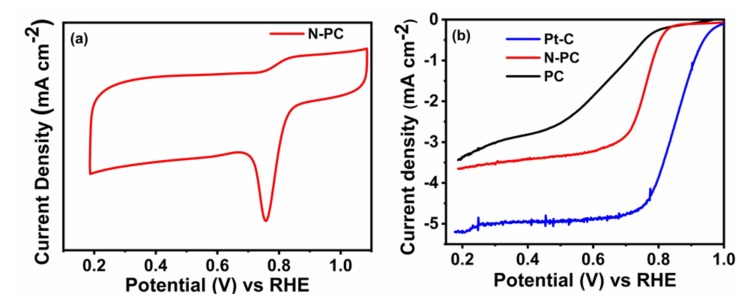
(**a**) CV curves of N-PC in O_2_ saturated; (**b**) LSV curves of PC, N-PC and Pt-C obtained from the RRDE measurement with rotating speed of 1600 rpm in 0.1 M KOH at a scan rate of 10 mV s^−1^.

**Figure 7:**
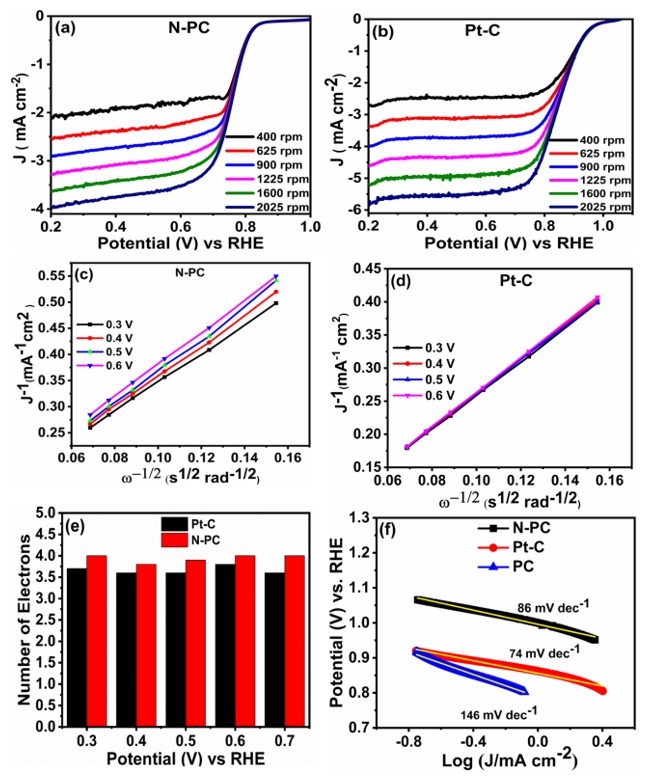
LSV curves of (**a**) N-PC and (**b**) Pt-C obtained from RRDE measurement with different rotation speeds in O_2_ saturated 0.1 M KOH at a scan rate of 10 mV s^−1^; Koutecky–Levich plots of (**c**) N-PC (**d**) Pt-C; (**e**) plot of potential vs number of electrons and (**f**) Tafel slopes of PC, N-PC and Pt-C.

The electrochemical ORR performances of PC, N-PC and Pt-C were examined through CV in 0.1 M KOH saturated with O_2_, as displayed in Figure 6a and Figure S4a. These measurements were conducted over a potential range from 0.2 to 1.2 V (vs RHE) with a scan rate of 10 mV s^−1^. It can be seen from Figure S4a, that the PC has barely any cathodic peaks for the O_2_ reduction. Meanwhile, the N-PC (Figure 6a) and Pt-C (Figure S4a) have distinguished cathodic peaks in the range between 0.7– 0.9 V (vs RHE) as a result of the presence of O_2_ reduction sites. Further, the LSV curves of PC, N-PC and Pt-C were recorded at an electrode rotation speed of 1600 rpm with a scan rate 10 mV s^−1^ in 0.1 M KOH saturated with O_2_, as depicted in Figure 6b. The N-PC has an onset potential of 0.83 V, which is less than that of Pt-C (0.98 V) and better than that of PC (0.78 V). The *E_1/2_* of N-PC is found to be at 0.76 V, whereas PC and commercial Pt-C exhibited corresponding values at 0.65 and 0.85 V, respectively. These results are credited solely to exposure of the pyridinic-N, pyrrolic-N and graphitic-N of N-PC for O_2_ reduction [13]. Table S1 shows that N-PC exhibited better electrocatalytic activity as compared to the recently reported electrocatalyst. The Figure 7a,b show that the LSV curve of N-PC and Pt-C were verified at various rotation speeds ranging from 400 to 2025 rpm at 10 mV s^−1^. The current density of N-PC is observed to be increasing at the increasing electrode rotation speed, which is due to the shortened diffusion distance at the electrode and O_2_ interface. The number of electrons transferred during ORR activity can be calculated through the Koutecky–Levich (K–L) equation [46]. 

Such a K–L plot derived from the plot between the inverse of the current density (J^−1^) and the inverse of square root of rotation rate. Figure 7c,d shows the K–L plot of N-PC and Pt-C catalysts. The K–L plot of N-PC shows good linearity between J^−1^ and with a potentials range of 0.3 to 0.6 V, showing similar electron transfer (closer to 4) at various potentials (Figure 7e). This result clearly indicates that N-PC follows first-order kinetics during ORRs and exhibits a dominant 4e^−^ electron transfer process (during the O_2_ conversion to H_2_O). The influence of catalyst loading on variation in the number of electrons was also studied and the results given in Figure S4b. The N-PC catalyst loading was varied from 0.025 mg cm^−2^ to 0.10 mg cm^−2^ on RRDE and the corresponding number of electrons transfer was evaluated. As the catalyst loading increases, the electron transfer number increases. The 0.025, 0.05, 0.075 and 0.1 mg cm^−2^ loading show ‘*n*’ values of 3.2, 3.6, 3.7 and 3.8, respectively. These results reveal that loading of the catalyst does not significantly influence the ‘n’ values [47]. To further get a deep understanding regarding the ORR kinetics of prepared catalysts, Tafel slopes of PC, N-PC and Pt-C were derived from LSV curves at low over potentials, as demonstrated in Figure 7f. The Tafel slope of N-PC (86 mV dec^−1^) is lower than that of PC (146 mV dec^−1^) or higher than commercial Pt-C (74 mV dec^−1^), indicating that N-PC catalyst has a faster electron transfer rate than PC during ORRs. The four electron pathway during O_2_ reduction on N-PC is described as follows. When incorporating heteroatoms in a porous carbon skeleton, the heteroatom (pyrrolic-N, pyridinic-N and graphitic-N) polarizes the adjacent carbon atom. Such carbon atoms emerge out of the skeleton and generate a chemical bond with O_2_. This is followed by a set of protons and electrons approaching the system to support the process of four electron reduction [48,49].

In order to determine the durability of N-PC and Pt-C during ORR activity, we carrier-out the chronoamperometry test for 10 h at 0.8 V in 0.1 M KOH solution with constant rotation speed of 1600 rpm (Figure S5). After a 10 h run, N-PC retained 86.5% of its initial current density, whereas Pt-C retained only 74.3% of its initial current density, clearly ratifying the extended electrochemical stability of N-PC with respect to commercial Pt-C.

#### 4.3.2. OER Activity and Durability

**Figure 8:**
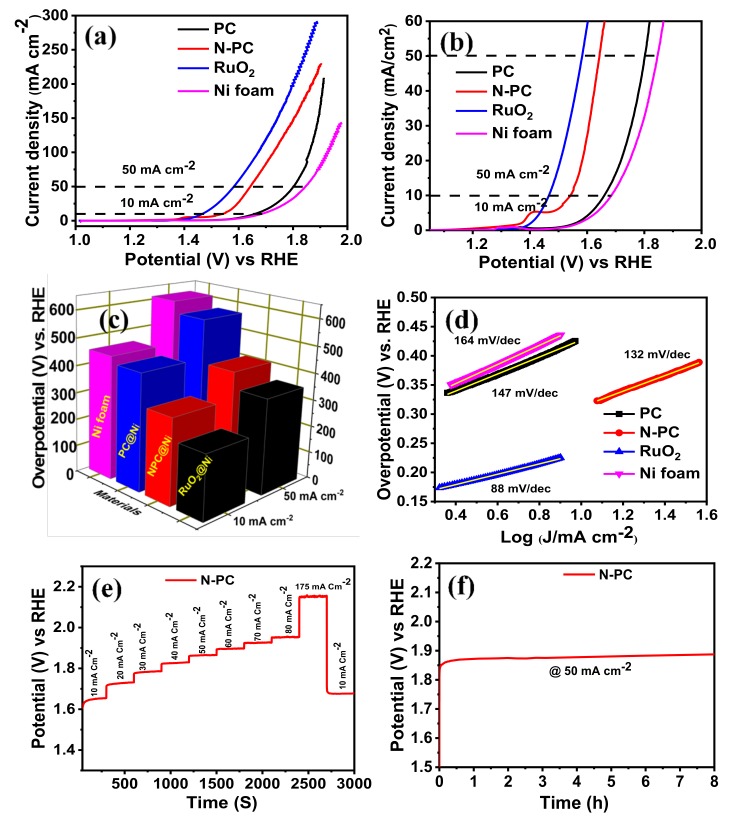
(**a**,**b**) Oxygen evolution reaction (OER)-LSV curves of PC@Ni, N-PC@Ni, RuO_2_@Ni and Ni foam in 1 M KOH at a scan rate 1 mV s^−1^ [with iR-correction]; (**c**) bar of prepared catalyst vs overpotential at 10 and 50 mA cm^−2^; (**d**) Tafel slopes of PC@Ni, N-PC@Ni, RuO_2_@Ni and Ni foam; (**e**) multiple-step chronopotentiometric of N-PC@Ni; (**f**) chronopotentiometric curve of N-PC@Ni at a constant of 50 mA cm^−2.^

OER activities of PC@Ni, N-PC@Ni, RuO_2_@Ni and Ni foam were identified through recording the LSV curves in 1 M KOH solution, as depicted in Figure 8a–c. The N-PC@Ni electrode exhibits an over potential of 314 mV at 10 mA cm^−2^ and 413 mV at 50 mA cm^−2^, whereas the PC@Ni and Ni electrode exhibits over potentials of 430 and 455 mV at 10 mA cm^−2^, and 572 and 614 mV at 50 mA cm^−2^, respectively_._ Still, the commercial RuO_2_@Ni electrode shows a lower over potential by 236 and 353 mV at 10 and 50 mA cm^−2^ as compared to N-PC. On the other hand, the obtained over potential of the N-PC@Ni electrode was significantly lower than N-doped carbon materials [50], N-doped carbon nanocages [51] and N and P co-doped mesoporous nanocarbon [35] reported in literatures (see Table S2). Further, catalytic kinetics of PC@Ni, N-PC@Ni, RuO_2_@Ni and Ni electrodes were evaluated by Tafel slope (Figure 8d). The Tafel slope of N-PC@Ni was 132 mV dec^−1^, which is less than the Ni electrode (164 mV dec^−1^) and PC@Ni electrode (147 mV dec^−1^), but it is higher than that of the RuO_2_@Ni (88 mV dec^−1^). This is because polarized carbon sites provided by N doping accelerate the OER performance in N-PC@Ni. CV curves were reordered in the non-faradaic region at various scan rates (5–50 mV s^−1^) to quantify the electrochemical double-layer capacitance (C_dl_) of catalysts (Figure S6a,b). The double-layer capacitance was evaluated from the slope value of the linear fitting curve (curve plotted between different of current density (*ΔJ* = *J*_anodic_ - *J*_cathodic_) vs RHE and scan rate). As given in Figure S6c, the PC and N-PC reveals the C_dl_ of 2.28 and 2.76 mF cm^−2^, respectively, suggesting the great number of active sites on the N-PC to support the catalytic activity.

The multiple-current chronopotentiometric curve was recorded for the N-PC@Ni electrode in 1 M KOH aqueous solution, as represented in Figure 8e. Initial potential starts at ~1.56 V (vs RHE) and thereafter, potential was kept at 1.64 V (vs RHE) at a current density of 10 mA cm^−2^. Afterward, potential decay was measured at each constant time interval of 300 s during applying the current density from 10 to 175 mA cm^−2^. By increasing current densities, the potentials of the N-PC@Ni electrode have risen gradually and are stable at each current density for 300 s. This confirms the superior mass transport property, conductivity and mechanical integrity of the N-PC@Ni electrode. When decreasing the current density by 10 mA cm^−2^ again, the 1.67 V was observed, which is almost similar to its initial potential. Such small variation in the potential is due to the peeling-off N-PC from Ni foam as a result of the quick generation/discharge of gas molecules at a higher current density. 

The long-term durability of the N-PC@Ni electrode was examined by chronopotentiometry measurements at a constant current density of 50 mA cm^−2^ for 8 h (Figure 8f). The initial potential of N-PC is 1.86 V at 50 mA cm^−2^, and after 8 h of electrolysis, this potential was slightly increased to 1.88 V, endorsing the better durability of N-PC@Ni.

#### 4.3.3. HER Activity and Durability 

**Figure 9:**
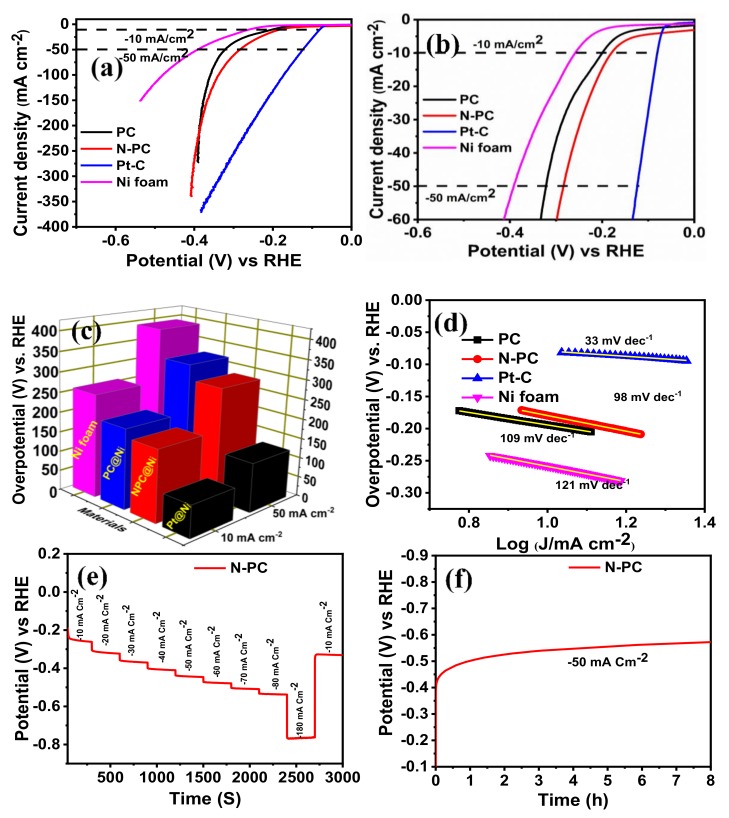
(**a**,**b**) Hydrogen evolution reaction (HER)-LSV curve of PC@Ni, N-PC@Ni, Pt-C@Ni, and Ni foam in 1 M KOH solution at a scan rate 1 mV s^−1^ [with iR-correction]; (**c**) bar of prepared catalyst vs overpotential at 10 and 50 mA cm^−2^; (**d**) Tafel slopes of PC@Ni, N-PC@Ni, Pt-C@Ni and Ni foam; (**e**) multiple-step chronopotentiometric of N-PC@Ni; (**f**) chronopotentiometric curve of N-PC@Ni at a constant of −50 mA cm^−2.^

The HER electrocatalytic activity of PC@Ni N-PC@Ni, Pt-C@Ni and Ni electrodes was evaluated in 1 M KOH solution. Figure 9a–c shows the comparative LSV curve of PC@Ni, N-PC@Ni, Pt-C@Ni and Ni electrodes, which demonstrate over potentials of 200, 179, 80 and 258 mV at −10 mA cm^−2^, and 321, 283, 124 and 392 mV at −50 mA cm^−2^, respectively. Among the aforesaid electrodes, Pt-C shows better HER activity owing to the existence of state-of-the-art Pt metal. On other hand, the N-PC@Ni electrode shows better HER activity with low over potential in relation to PC@Ni and the Ni electrode, representing the effect of N doping. According to Table S3, the N-PC@Ni electrode has lower over potential when compared to various catalysts reported in the References. The kinetics of HER on the PC@Ni, N-PC@Ni, Pt-C@Ni and Ni electrodes has been calculated from Tafel slopes (Figure 9d). The Tafel slope of N-PC@Ni was 98 mV dec^−1^, which is lower than that of PC@Ni (109 mV dec^−1^) and Ni foam (121 mV dec^-1^). Otherwise, it is higher than Pt-C@Ni (33 mV dec^−1^). Suggestively, efficient HER kinetics are provided by N-PC compared to PC@Ni and Ni. The stability of the N-PC@Ni electrode was also evaluated using multi-step chronopotentiometry in 1 M KOH. Figure 9e displays the multi-step chronopotentiometry curve of this N-PC@Ni electrode. The potential decay was measured by applying current density from −10 to −180 mA cm^−2^ with step size of −10 mA cm^−2^. For each current density, the holding time was 300 s. The obtained result ratifies that N-PC@Ni has a low tendency to peeling-off during HER.

Long-term durability of N-PC@Ni during HERs was examined by the chronopotentiometry method at −50 mA cm^−2^ for 8 h in 1 M KOH. The chronopotentiometry curve (Figure 9f) confirms that the N-PC@Ni electrode has better stability in HER. From the aforementioned findings, it would be concluded that N-PC is a swatch candidate towards tri-functional electrochemical reactions which include ORRs, OERs and HERs. The N doping, which alters the electron density in the carbon framework, and the porous structures of N-PC, cumulatively endow the better electrochemical activities and stabilities.

## 5. Conclusions

In summary, we introduced the synthesis of PC and N-PC from low-cost GSB via a solvent-free method. Formation and N doping in N-PC was confirmed by Field Emission Scanning Electron Microscope (FESEM), energy-dispersive analysis X-ray (EDAX), Raman and X-ray photoelectron spectroscopy (XPS) techniques. N-PC revealed robust, tri-functional, catalytic performance towards HERs, OERs and ORRs in alkaline electrolyte. The relatively large and self-organized porous structures of N-PC were observed by FE-SEM and BET. The N-PC displays ORR, OER and HER activities comparable to Pt-C and RuO_2_, but better to PC in alkaline electrolyte. Its long-term stability and four-electron transfer tendency were superior compared to that of Pt-C and RuO_2_. 

The mechanical integrity and mass-transfer properties of N-PC were also better during OER and HER processes. The strategy adopted in this present work and investigation demonstrated that the structure of N-PC can be further tuned with varying the heteroatom content and type. If efforts are put further, this strategy might open up new possibilities for the fabrication of efficient porous carbon architectures from bio-wastes for energy applications, such as water splitting and a metal–air battery.

## Supplementary Materials

The following are available online at https://www.mdpi.com/2079-4991/10/1/76/s1, Figure S1: (a-d) SEM image elemental mapping of C, N, and O for N-PC; Figure S2:Raman spectrum of PC with a deconvolution of the D/G spectral region; Figure S3: Deconvoluted XPS spectra of PC (a) C1s (b) O1s; Figure S4: (a) CV curves of PC and Pt-C in O_2_ saturated (b) Effect of the catalyst loading on the number of electrons transferred; Figure S5: Long term durability of N-PC and Pt-C in 0.1 M KOH; Figure S6: CV curves of (a) PC and (b) N-PC with different scan rate from 5–25 mV S^–^^1^; (c) linear fit of *ΔJ*/2 Vs scan rate, Table S1: Comparison of ORR performance for N-PC with other Bio-derived activated carbon; Table S2: Comparison of OER performance for N-PC@Ni with other N-doped carbon materials; Table S3: Comparison of HER performance for N-PC@Ni with other N-doped carbon materials.
