# Supplementary material for: Nitrogen-Doped Porous Carbon Derived from Biomass Used as Trifunctional Electrocatalyst toward Oxygen Reduction, Oxygen Evolution and Hydrogen Evolution Reactions
Source: Nanomaterials (Basel). 2019 Dec 31;10(1):76. doi: 10.3390/nano10010076 (PMC7022927; doi:10.3390/nano10010076)
Supplement: Supplementary file 1 [file nanomaterials-10-00076-s001.pdf]

nanomaterials

Supplementary Materials

Nitrogen-Doped Porous Carbon Derived from Biomass Used as Trifunctional Electrocatalyst toward Oxygen Reduction, Oxygen Evolution and Hydrogen Evolution Reactions

Chinnusamy Sathiskumar [1], Shanmugam Ramakrishnan [2], Mohanraj Vinothkannan [2] Ae Rhan Kim [3], Srinivasan Karthikeyan, [4] and Dong Jin Yoo [2,5,*]

[1] Centre for Nano and Soft Matter Science, (CeNS), Jalahalli, Bengaluru-560013, India; sathischem85@gmail.com
[2] R&D Education center for whole life cycle R&D of fuel cell systems, Jeonbuk National University, Jeollabuk-do 54896, Korea; rammtech09@gmail.com (S.R.); vinothkannanram@gmail.com (M.V.)
[3] R&D Center for CANUTECH, Business Incubation Center, Department of Bioenvironmental Chemistry, Jeonbuk National University, Jeollabuk-do 54896, Korea; kimaerhan@jbnu.ac.kr
[4] Department of Chemistry, Chikkanna Government Arts College, Tirupur-641502, Tamil Nadu, India; environkarthi@gmail.com
[5] Department of Life Science, Graduate School of Department of Energy Storage/Conversion Engineering, and Hydrogen and Fuel Cell Research Center, Jeonbuk National University, Jeollabuk-do 54896, Korea
* Correspondence: djyoo@jbnu.ac.kr (D.J.Y); Tel.: +82-(0)63-270-3608. Fax: +82- (0)63-270-3909.

Measurement of the Number of Electron and Koutecky-Levich (K-L) plot

The linear sweep voltammetry (LSV) technique was conducted with an RDE loaded with as-prepared samples at different rotation rates to investigate the electrode kinetics toward ORR. The overall electron transfer number (n) per oxygen molecule in a typical ORR process on different electrodes can be calculated using the Koutecky-Levich (K-L) equation as follows.

$$1/j = 1/j_k + 1/0.62nFC(D)2/3\omega 1/2v\text{-}1/6) \qquad (1)$$

where j and j_K are the measured and kinetic-limited current density respectively. j_K is assumed to be a constant at a certain potential. F is the Faraday constant; n is the number of electrons transferred per oxygen molecule; D is the diffusion coefficient of O_2 in 0.1M KOH; C is the bulk concentration of O_2 (1.2 x 10-3 mol/liter); ν is the kinematic viscosity of electrolyte (0.01 cm2 /s); ω is the rotation in rpm; and k is the electron transfer rate constant. From the corresponding K-L plots, the data exhibited good linearity. Then, n can be calculated as

$$n = 4\frac{I_d}{I_d + I_r/N} \qquad (2)$$

where I_r is the ring current, I_d is the disk current, and N is the current collection efficiency of the Pt ring, which was determined to be 0.41.

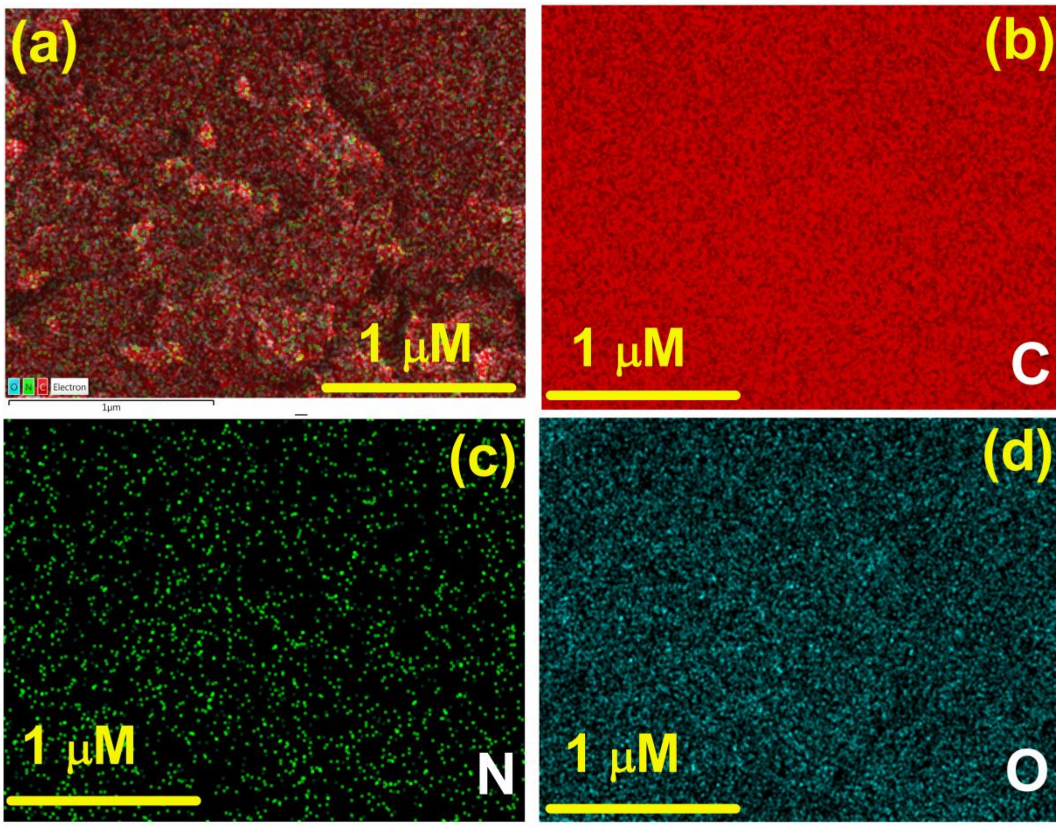

Figure S1. (**a**–**d**) SEM image elemental mapping of C, N, and O for N-PC.

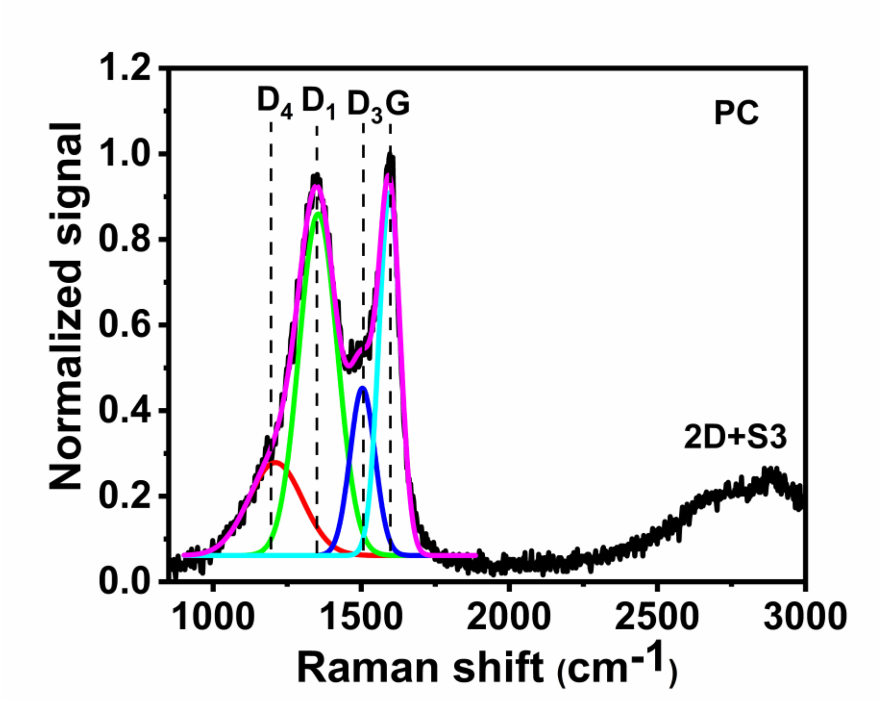

Figure S2. Raman spectrum of PC with a deconvolution of the *D*/*G* spectral region.

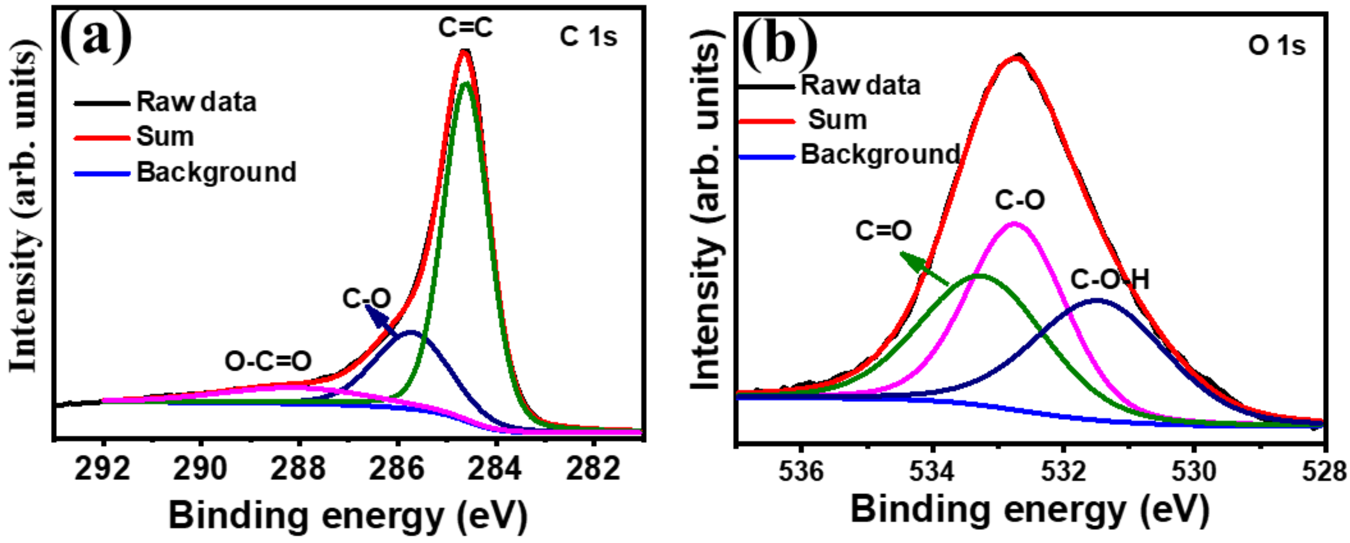

Figure S3. Deconvoluted XPS spectra of (a) C 1s and (b) O 1s for PC.

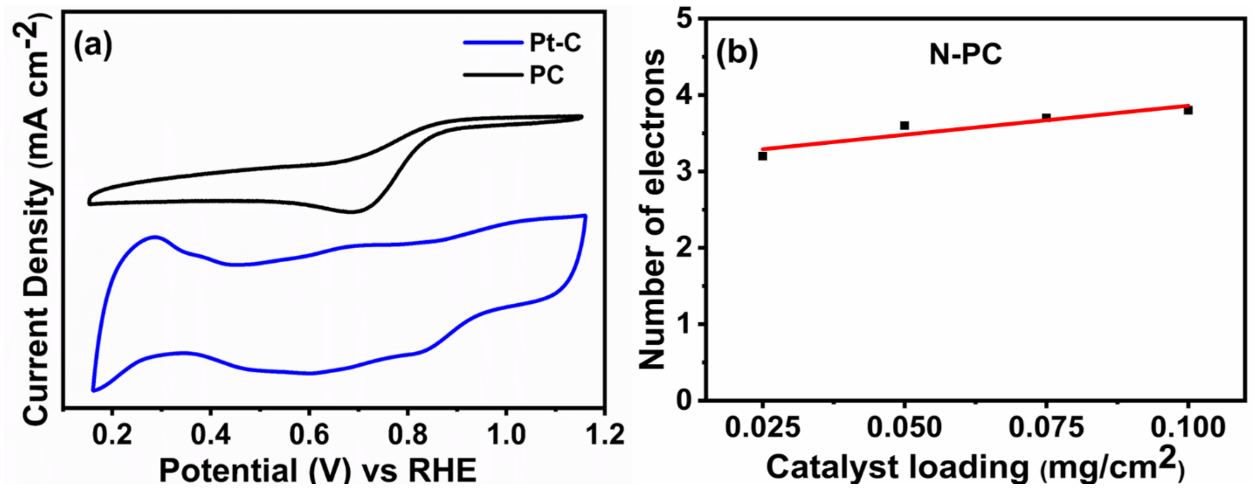

Figure S4. (**a**) CV curves of PC and Pt-C in O_2 saturated (**b**) Effect of the catalyst loading on the number of electrons transferred.

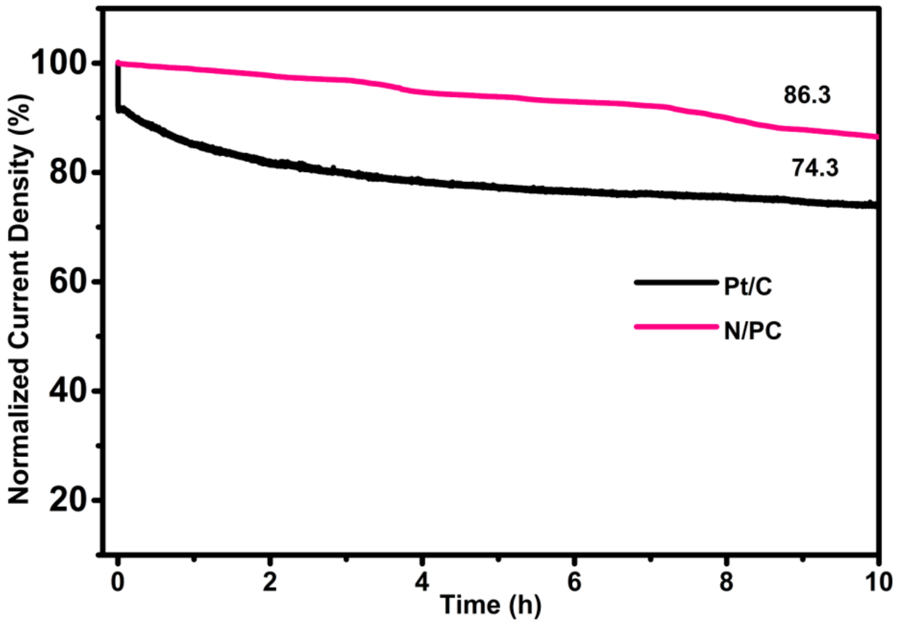

Figure S5. Long term durability of N-PC and Pt-C in 0.1 M KOH.

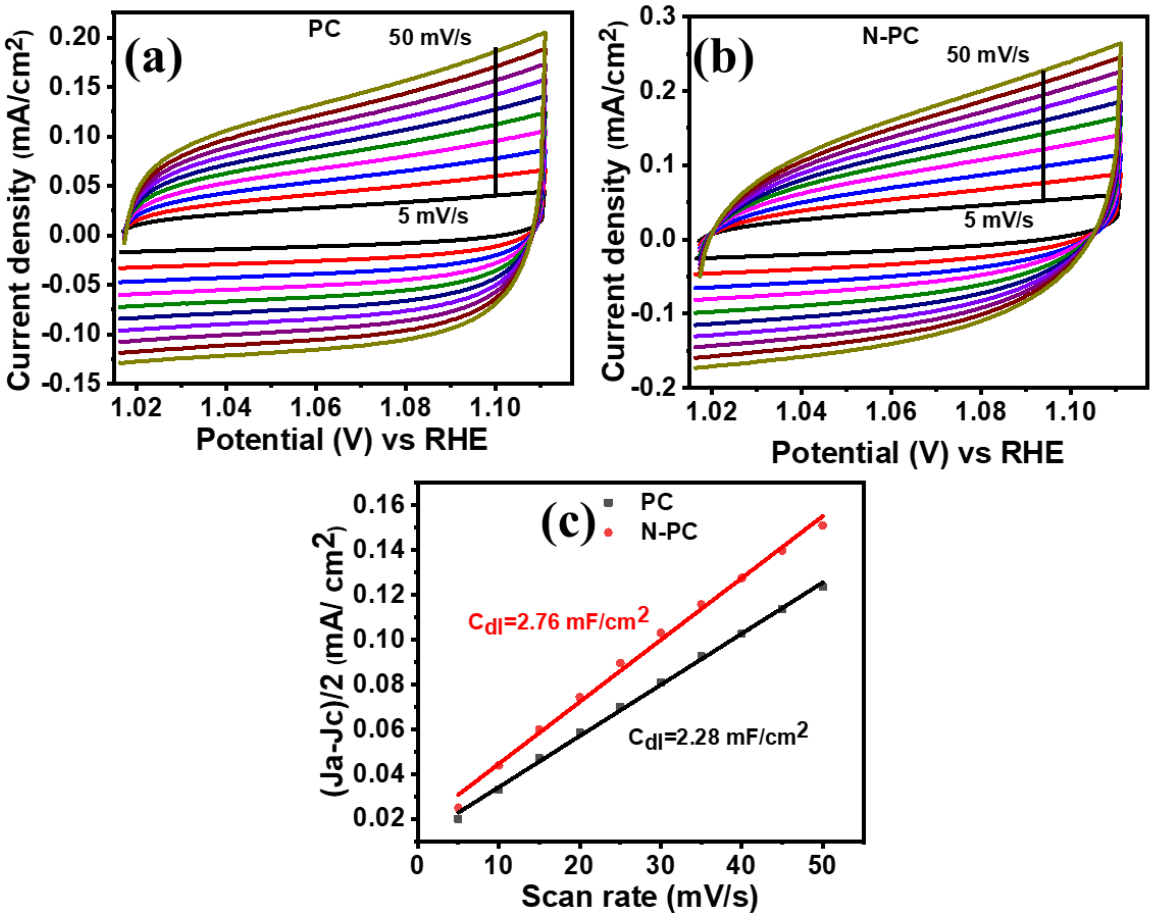

Figure S6. CV curves of (**a**) PC and (**b**) N-PC with different scan rate from 5–25 mV S^{-1}; (**c**) linear fit of ΔJ/2 Vs scan rate.

Table S1. Comparison of ORR performance for N-PC with other Bio-derived activated carbon.

The precursor for Carbon source	Sample Name	Onset potential vs RHE (V)	Electrolyte	References
Golden shower	N/PC	0.83	0.1M KOH	This work
Banana peel	BPPC-MO50	0.9259	0.1M KOH	[1]
Banana peel	N-BPDC-H_3PO_4-1000	0.92	0.1M KOH	[2]
wastesoybean dregs	3D-NCN	0.82	0.1M KOH	[3]
Kidney bean	KB 350Z900	0.90	KOH	[4]
Orange peel	Pt/OP-AC	0.84	HClO4	[5]
Soybean	Fe/CeSOYB	0.84	1 M KOH	[6]

Table S2. Comparison of OER performance for N-PC@Ni with other N-doped carbon materials.

Sample Name	Overpotential at 10 mA cm-2 (mV)	Electrolyte	References
N/PC@Ni	314	1 M KOH	This work
N/C	380	KOH-pH-13	[7]
N-CCs	460	0.1 M NaOH	[8]
NPMC	390	0.1 M KOH	[9]
Fe/N-CNSs	498	0.1 M KOH	[10]
NMWNT	320	1 M NaOH	[11]
N-GRW	360	1M KOH	[12]
Ni foam/N-NTs/Ni(OH)	254	1M KOH	[13]
N-doped CNTs	370	1M KOH	[14]
NCF-900	340	1 M KOH	[15]

Table S3. Comparison of HER performance for N-PC@Ni with other N-doped carbon materials.

Sample Name	Overpotential at 10 mA cm-2 (mV)	Electrolyte	References
N/PC@Ni	179	1 M KOH	This work
NMWNT	235	0.1 M NaOH	[11]
C_3N_4@NG	240	0.5 M $H2SO_4$	[17]
N,P-graphene-1	422	0.5 M $H2SO_4$	[18]
Co-N-GA	232	1.0 M KOH	[19]
SHG	230	0.1 M KOH	[20]
Ni-NC700	301	0.1 M KOH	[21]
FeNi+NPC	340	0.1 M KOH	[16]
N,S-CN	380	1 M KOH	[22]
FeCo+NPC	450	0.1 M KOH	[16]

References

1. Wang, Y.; Zhu, M.; Li, Y.; Zhang, M.; Xue, X.; Shi, Y.; et al. Heteroatom-doped porous carbon from methyl orange dye wastewater for oxygen reduction. *Green Energy Environ.* **2018,** 3, 172–178.
2. Kong, D.; Liu, L.; Yuan, W.; Xie, A.; Shen, Y. Facile synthesis and excellent catalytic performance of nitrogen-doped porous carbons derived from banana peel towards oxygen reduction reaction. *Mater Res. Bull.* **2018,** 103, 63–69.
3. Chen, Y.; Wang, H.; Ji, S.; Lv, W.; Wang, R. Harvesting a 3D N-doped carbon network from waste bean dregs by ionothermal carbonization as an electrocatalyst for an oxygen reduction reaction. *Materials (Basel)* **2017**, 10.
4. Dhelipan, M.; Arunchander, A,; Sahu, A.K.; Kalpana, D. Activated carbon from orange peels as supercapacitor electrode and catalyst support for oxygen reduction reaction in proton exchange membrane fuel cell. *J. Saudi Chem. Soc.* **2017,** 21, 487–494.

5. Guo, C.Z.; Liao, W.L.; Chen, C.G. Design of a non-precious metal electrocatalyst for alkaline electrolyte oxygen reduction by using soybean biomass as the nitrogen source of electrocatalytically active center structures. *J. Power Sources* **2014,** 269, 841–847.
6. Wang, Y.; Zhu, M.; Wang, G.; Dai, B.; Yu, F.; Tian, Z.; et al. Enhanced Oxygen Reduction Reaction by In Situ Anchoring Fe2N Nanoparticles on Nitrogen-Doped Pomelo Peel-Derived Carbon. *Nanomaterials* **2017**, 7, 404.
7. Zhao, Y.; Nakamura, R.; Kamiya, K.; Nakanishi, S.; Hashimoto, K. Nitrogen-doped carbon nanomaterials as non-metal electrocatalysts for water oxidation. *Nat. Commun.* **2013**, 4, 1–7.
8. Jia, N.; Weng, Q.; Shi, Y.; Shi, X.; Chen, X.; Chen, P.; et al. N-doped carbon nanocages: Bifunctional electrocatalysts for the oxygen reduction and evolution reactions. *Nano Res.* **2018,** 11, 1905–1916.
9. Zhang, J.; Zhao, Z.; Xia, Z.; Dai, L. A metal-free bifunctional electrocatalyst for oxygen reduction and oxygen evolution reactions. *Nat. Nanotechnol.* **2015,** 10, 444–452.
10. Jia, J.; Liu, Z.; Han, F.; Kang, G.J.; Liu, L.; Liu, J.; et al. The identification of active N species in N-doped carbon carriers that improve the activity of Fe electrocatalysts towards the oxygen evolution reaction. *RSC Adv.* **2019,** 9, 4806–4811.
11. Davodi, F.; Tavakkoli, M.; Lahtinen, J.; Kallio, T. Straightforward synthesis of nitrogen-doped carbon nanotubes as highly active bifunctional electrocatalysts for full water splitting. *J Catal.* **2017,** 353, 19–27.
12. Yang, H.B.; Miao, J.; Hung, S.F.; Chen, J.; Tao, H.B.; Wang, X.; et al. Identification of catalytic sites for oxygen reduction and oxygen evolution in N-doped graphene materials: Development of highly efficient metal-free bifunctional electrocatalyst. *Sci Adv.* **2016,** 2, e1501122.
13. Wu, J.; Subramaniam, J.; Liu, Y.; Geng, D.; Meng, X. Facile assembly of Ni(OH)2 nanosheets on nitrogen-doped carbon nanotubes network as high-performance electrocatalyst for oxygen evolution reaction. J. Alloys Compd. **2018**, 731, 766–773.
14. Xia, B.Y.; Yan. Y.; Li, N.; Wu, H.B.; Lou, X.W.D.; Wang, X. A metal-organic framework-derived bifunctional oxygen electrocatalyst. *Nat. Energy* **2016,** 1, 1–8.
15. Yang, X.; Li, K.; Cheng, D.; Pang, W.L.; Lv, J.; Chen, X.; et al. Nitrogen-doped porous carbon: Highly efficient trifunctional electrocatalyst for oxygen reversible catalysis and nitrogen reduction reaction. *J. Mater Chem. A* **2018,** 6, 7762–7769.
16. Zhong, H.X.; Wang, J.; Zhang, Q.; Meng, F.; Bao, D.; Liu, T.; et al. In Situ Coupling FeM (M = Ni, Co) with Nitrogen-Doped Porous Carbon toward Highly Efficient Trifunctional Electrocatalyst for Overall Water Splitting and Rechargeable Zn-Air Battery. *Adv Sustain. Syst.* **2017,** 1, 1700020.
17. Zheng, Y.; Jiao, Y.; Zhu, Y.; Li, L.H.; Han, Y.; Chen, Y.; et al. Hydrogen evolution by a metal-free electrocatalyst. *Nat. Commun.* **2014**, 5, 1–8.
18. Zheng, Y.; Jiao, Y.; Li, L.H.; Xing, T.; Chen, Y.; Jaroniec, M.; et al. Toward design of synergistically active carbon-based catalysts for electrocatalytic hydrogen evolution. *ACS Nano* **2014,** 8, 5290–5296.
19. Zhu, Z.; Yang, Y.; Guan, Y.; Xue, J.; Cui, L. Construction of a cobalt-embedded nitrogen-doped carbon material with the desired porosity derived from the confined growth of MOFs within graphene aerogels as a superior catalyst towards HER and ORR. *J. Mater. Chem. A* **2016,** 4, 15536–15545.
20. Hu, C.; Dai, L. Multifunctional Carbon-Based Metal-Free Electrocatalysts for Simultaneous Oxygen Reduction, Oxygen Evolution, and Hydrogen Evolution. *Adv. Mater.* **2017**, 29, 1604942.
21. Devi, B.; Koner, R.R.; Halder, A. Ni(II)-Dimeric Complex-Derived Nitrogen-Doped Graphitized Carbon-Encapsulated Nickel Nanoparticles: Efficient Trifunctional Electrocatalyst for Oxygen Reduction Reaction, Oxygen Evolution Reaction, and Hydrogen Evolution Reaction. *ACS Sustain. Chem. Eng.* **2019,** 7, 2187–2199.
22. Qu, K.; Zheng, Y.; Zhang, X.; Davey, K.; Dai, S.; Qiao, S.Z. Promotion of Electrocatalytic Hydrogen Evolution Reaction on Nitrogen-Doped Carbon Nanosheets with Secondary Heteroatoms. *ACS Nano* **2017,** 11, 7293–7300.
